# Relationships between Inflammation, Adiponectin, and Oxidative Stress in Metabolic Syndrome

**DOI:** 10.1371/journal.pone.0045693

**Published:** 2012-09-19

**Authors:** Shu-Ju Chen, Chi-Hua Yen, Yi-Chia Huang, Bor-Jen Lee, Simon Hsia, Ping-Ting Lin

**Affiliations:** 1 Department of Health Food, Chung Chou University of Science and Technology, Changhua, Taiwan; 2 Department of Family and Community Medicine, Chung Shan Medical University Hospital, Taichung, Taiwan; 3 School of Medicine, Chung Shan Medical University, Taichung, Taiwan; 4 Center for Education and Research on Geriatrics and Gerontology, Chung Shan Medical University, Taichung, Taiwan; 5 School of Nutrition, Chung Shan Medical University, Taichung, Taiwan; 6 Department of Nutrition, Chung Shan Medical University Hospital, Taichung, Taiwan; 7 The Intensive Care Unit, Taichung Veterans General Hospital, Taichung, Taiwan; 8 Department of Nutrition and Institute of Biomedical Nutrition, HungKuang University, Taichung, Taiwan; Statens Serum Institute, Denmark

## Abstract

Metabolic syndrome (MS) represents a cluster of physiological and anthropometric abnormalities. The purpose of this study was to investigate the relationships between the levels of inflammation, adiponectin, and oxidative stress in subjects with MS. The inclusion criteria for MS, according to the Taiwan Bureau of Health Promotion, Department of Health, were applied to the case group (n = 72). The control group (n = 105) comprised healthy individuals with normal blood biochemical values. The levels of inflammatory markers [high sensitivity C-reactive protein (hs-CRP) and interleukin-6 (IL-6), adiponectin, an oxidative stress marker (malondialdehyde), and antioxidant enzymes activities [catalase (CAT), superoxide dismutase (SOD), and glutathione peroxidase (GPx)] were measured. Subjects with MS had significantly higher concentrations of inflammatory markers and lower adiponectin level, and lower antioxidant enzymes activities than the control subjects. The levels of inflammatory markers and adiponectin were significantly correlated with the components of MS. The level of hs-CRP was significantly correlated with the oxidative stress marker. The IL-6 level was significantly correlated with the SOD and GPx activities, and the adiponectin level was significantly correlated with the GPx activity. A higher level of hs-CRP (≥1.00 mg/L), or IL-6 (≥1.50 pg/mL) or a lower level of adiponectin (<7.90 µg/mL) were associated with a significantly greater risk of MS. In conclusion, subjects suffering from MS may have a higher inflammation status and a higher level of oxidative stress. A higher inflammation status was significantly correlated with decreases in the levels of antioxidant enzymes and adiponectin and an increase in the risk of MS.

## Introduction

Metabolic syndrome (MS) represents a clustering of physiological and anthropometric abnormalities [1] and is recognized as a significant risk factor for cardiovascular disease and type II diabetes [2]. The Third National Health and Nutrition Examination Survey (NHANES 1988–1994) reported that more than 20% of the adult population in the US suffered from MS [3], [4]. A recent NHANES (2003–2006) reported that the prevalence rates of MS were 35.1% in men and 32.6% in women [5]. In Taiwan, a report from the Nutrition and Health Survey in Taiwan (NAHSIT) conducted during 1993–1996 observed that the prevalence rates of MS were 13.6% in men and 26.4% in women. A recent report from NAHSIT (2005–2008) reported that the prevalence rates of MS had increased to 25.5% in men and 31.5% in women [6]. The markers of MS, including insulin resistance, type II diabetes, hypertension, dyslipidemia, and visceral obesity, may increase oxidative stress [7]–[9] and reduce antioxidant defenses [10]–[12]. Increases in oxidative stress contribute to impaired vascular function, inflammation, thrombosis, and atherosclerosis and ultimately give rise to vascular disease [13].

The proinflammatory status may lead to the clinical and biochemical manifestations of MS [14]. In clinical studies, the levels of high sensitivity C-reactive protein (hs-CRP) and interleukin-6 (IL-6) are commonly used as inflammatory markers that contribute the early stages of coronary artery disease [15]. hs-CRP is a product of hepatic inflammatory and is under the regulation of IL-6 [16], [17]; IL-6 is a messenger cytokine (pro-inflammatory cytokine) that is secreted by macrophages and smooth muscle cells in atherosclerotic lesions. Adiponectin is an anti-inflammatory marker that is potentially antiatherogenic and is secreted in abundance by adipocytes in normal subjects [14], [18]. Recent clinical studies suggest that low-grade inflammation might play an important role in the pathobiology of MS [19], [20]. It is worth knowing the levels of inflammatory markers and adiponectin that are associated with increased risk of MS. In addition, studies concerning the associations of the inflammation status and adiponectin with oxidative stress are limited. Therefore, the purpose of this study was to investigate the relationship between inflammatory markers (hs-CRP and IL-6), adiponectin, oxidative stress, and the components of MS. We also calculated the odds ratio of MS based on the levels of the inflammatory markers and adiponectin.

## Materials and Methods

### Subjects

The current study was designed as a case-control study. We expected the differences in the mean levels of hs-CRP between the case and control groups to be 1.0±1.5 mg/L; therefore, the desired power was set at 0.8 to detect a true effect. For α = 0.05, this power yielded a minimal simple size of 37 participants in each group. Subjects with MS (case group, n = 72) were recruited from the Department of Family and Community Medicine of Chung Shan Medical University Hospital in Taiwan. The inclusion criteria for MS in adults were based on the guidelines of the Bureau of Health Promotion, Department of Health, Taiwan (2007). Subjects were considered to have MS if they had three of the following five characteristics: (1) abdominal obesity (waist circumference ≥90 cm in men and ≥80 cm in women), (2) impaired fasting glucose (≥5.6 mmol/L), (3) hypertriglyceridemia (≥1.7 mmol/L), (4) low high-density lipoprotein cholesterol (HDL-C <1.0 mmol/L in men and <1.3 mmol/L in women), and (5) increased blood pressure (systolic blood pressure ≥130 mmHg and diastolic blood pressure ≥85 mmHg). Subjects using antidiabetic, antihypertensive, and lipid-lowering medications were considered to have elevated fasting blood glucose, elevated blood pressure, and dyslipidaemia, respectively. Case subjects who were on statin therapy were excluded. Control subjects (n = 105) were recruited from the physical examination unit of the hospital, and exhibited normal blood biochemical values, including fasting glucose <5.6 mmol/L, blood urea nitrogen <7.9 mmol/L, creatinine <123.8 µmol/L, alkaline phosphates <190 U/L, glutamic oxaloacetic transaminase <35 U/L, and glutamic pyruvate transaminase <45 U/L. Control subjects did not have any illnesses or a history of gastrointestinal disorders, hypertension, hyperlipidemia, liver or renal disease, diabetes, or other metabolic disease. Subjects who were taking antioxidant vitamin supplements were excluded. This study was approved by the Institutional Review Board of Chung Shan Medical Hospital in Taiwan and written informed consent was obtained from each subject.

The age, blood pressures, drinking, and smoking habits of the subjects were recorded. The blood pressure was measured in each patient after resting for at least 5 min. The body weight, height, waist, and hip circumferences were measured, and the body mass index (kg/m^2^) and the waist to hip ratio were then calculated.

### Blood collection and biochemical measurement

Fasting venous blood samples (15 mL) were obtained to estimate the hematological parameters and vitamin status. Blood specimens were collected in vacutainer tubes with or without EDTA as an anticoagulant as needed. Serum and plasma were prepared and then frozen (−80°C) for storage until analysis. Blood lipid profiles [i.e., total cholesterol (TC), triacylglycerol, LDL-C, and HDL-C], and hs-CRP concentrations were measured using an automated biochemical analyzer (Hitachi-7180E, Tokyo, Japan). The quantitative measurements of the serum levels of IL-6 (eBioscience, San Diego, CA, USA) and adiponectin (BioVendor, Brno, Czech Republic) were performed using commercially available enzyme-linked immunosorbent assay kits, and the absorbances were measured at 450 nm (BIO-RAD Microplate Reader Model 680, Hercules, CA, USA).

The plasma malondialdehyde (MDA) level was determined using the thiobarbituric acid reactive substances (TBARS) method [21]. Red blood cells (RBCs) were diluted with 25x sodium phosphate buffer for the SOD and GPx measurements and 250x sodium phosphate buffer for the CAT measurement. The methods for measuring the CAT, SOD and GPx activities in RBCs have previously been described [22]–[24]; these measurements were performed spectrophotometrically at 240 nm, 325 nm and 340 nm, respectively. The RBC protein content was determined using the BCA kit (Thermo, Rockford, IL, USA), which is based on the biuret reaction. The antioxidant enzymes activity levels were expressed as units/mg protein. All analyses were performed in duplicate and repeated measurements of the same sample varied by less than 10%. The analyses of the plasma MDA and the antioxidant enzymes activities were completed within 7 days of blood collection.

### Statistical analyses

The data were analyzed using SigmaPlot software (version12.0, Systat, San Jose, CA, USA). The distribution of variables was evaluated using the Shapiro-Wilk test. The differences in the demographic and hematological characteristics between the case and control groups were analyzed using Student's t-test or the Mann-Whitney rank sum test. For categorical response variables, differences between the two groups were assessed using the Chi-square test or Fisher's exact test. Pearson's correlation or Spearman rank order correlation analyses were performed to examine the correlations between the levels of inflammatory markers, adiponectin, oxidative stress, antioxidant enzymes activities, and the components of MS. Adjusted odds ratios (ORs) with 95% confidence intervals (CI) for MS were calculated from the logistic regression models based on the levels of inflammatory markers and adiponectin. The data are expressed as the means ± standard deviations (SD). The results were considered statistically significant at *p*<0.05.

## Results

Table 1 shows the demographic data and the health characteristics of the subjects. The subjects in the case group had significantly higher values for systolic blood pressure, diastolic blood pressure, body mass index, waist circumference, waist to hip ratio, hematological parameters (i.e., fasting glucose, TG, LDL-C, and TC/HDL-C), and lower HDL-C level than the control group.

**Table 1:** Characteristics of subjects.

	Case (n = 72)	Control (n = 105)	*p* values
Male/Female (n)	43/29	52/53	0.24
Age (y)	53.3±11.6^1^	52.0±8.1	0.06
Systolic blood pressure (mmHg)	141.9±11.8	118.8±16.8	<0.01
Diastolic blood pressure (mmHg)	88.5±10.3	77.7±9.8	<0.01
Body mass index (kg/m^2^)	29.1±5.8	24.4±3.5	<0.01
Waist circumference (cm)	96.0±12.4	80.6±13.6	<0.01
Waist to hip ratio	0.93±0.07	0.85±0.11	<0.01
Fasting glucose (mmol/L)	7.5±2.6	5.2±1.1	<0.01
TC (mmol/L)	4.9±1.0	5.1±0.9	0.19
TG (mmol/L)	1.9±0.9	1.3±0.6	<0.01
LDL-C (mmol/L)	3.2±0.9	2.9±0.7	0.04
HDL-C (mmol/L)	1.2±0.3	1.4±0.4	<0.01
TC/HDL-C	4.4±1.2	3.9±1.2	<0.01
Current smoker^2^, n (%)	15 (20.8%)	13 (12.4%)	0.19
Drink alcohol^3^, n (%)	8 (11.1%)	15 (14.3%)	0.20
Exercise^4^, n (%)	40 (55.6%)	63 (60.0%)	0.66

1 Mean ± SD. ^2^Current smoker: individuals currently smoking one or more cigarettes per day. ^3^Drink alcohol: individuals drinking one or 1 more drink regularly. ^4^Excerise: individuals exercise regularly at least 3 times every week. HDL-C, high-density lipoprotein-cholesterol; LDL-C, low density lipoprotein-cholesterol; TC, total cholesterol; TG, triglyceride.

**Figure 1:**
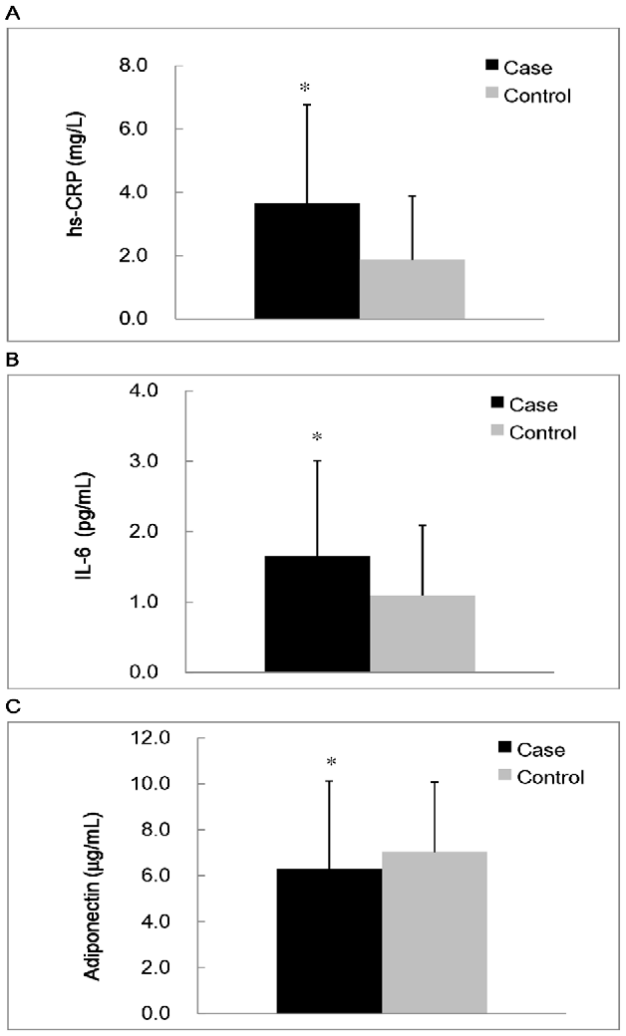
Concentrations of inflammatory makers (A, B) and adiponectin (C). ^*^Values were significantly different between the case and control groups; *p*<0.05. hs-CRP, high sensitivity C-reactive protein; IL-6, interleukin-6.

**Figure 2:**
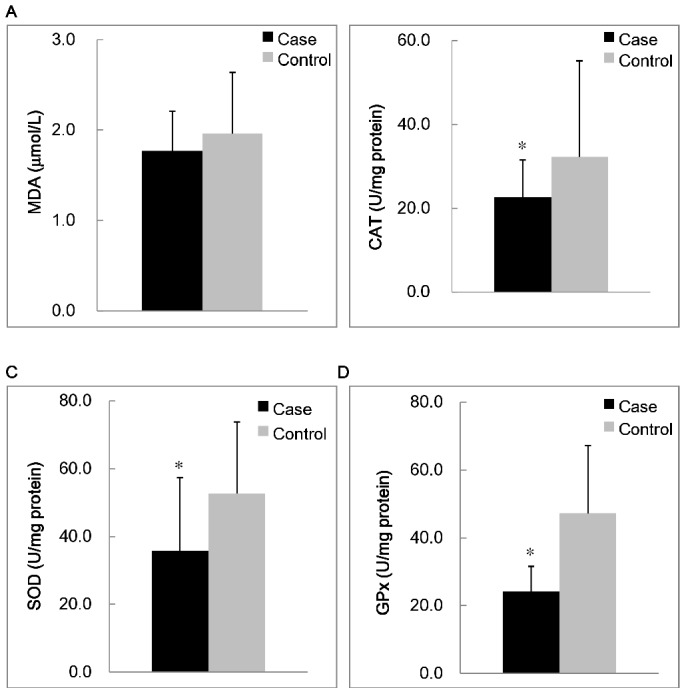
Concentrations of lipid peroxidation marker (A) and antioxidant enzymes activities (B to D). ^*^Values were significantly different between the case and control groups; *p*<0.05. CAT, catalase activity; GPx, glutathione peroxidase; MDA, malondialdehyde; SOD, superoxide dismutase.

The concentrations of inflammatory markers, adiponectin, and an oxidative stress are shown in Figure 1 and Figure 2. The subjects in the case group had significantly higher levels of hs-CRP (*p* = 0.01) and IL-6 (*p* = 0.03), and lower level of adiponectin (*p*<0.01) than the control group. With respect to antioxidant enzymes activities, the subjects with MS had significantly lower CAT (*p* = 0.02), SOD (*p*<0.01), and GPx activities (*p*<0.01) than the subjects in the control group. However, the MDA level was not significantly different between two groups.

The correlations between the levels of inflammatory markers, adiponectin, and the components of MS are shown in Table 2. The level of hs-CRP was significantly correlated with the values of diastolic blood pressure (*r* = 0.26, *p*<0.01), waist circumferences (*r* = 0.47, *p*<0.01), fasting glucose (*r* = 0.27, *p*<0.01), TG (*r* = 0.20, *p* = 0.01), and HDL-C (*r* = −0.26, *p*<0.01). The level of IL-6 was significantly correlated with the values of diastolic blood pressure (*r* = 0.20, *p* = 0.01), waist circumferences (*r* = 0.33, *p*<0.01), and HDL (*r* = −0.22, *p*<0.01). The level of adiponectin was significantly correlated with the values of diastolic blood pressure (*r* = −0.28, *p*<0.01), waist circumferences (*r* = −0.30, *p*<0.01), fasting glucose (*r* = −0.25, *p*<0.01), TG (*r* = −0.32, *p*<0.01), HDL-C (*r* = 0.41, *p*<0.01), hs-CRP (*r* = −0.24, *p*<0.01), and IL-6 (*r* = −0.11, *p* = 0.06).

**Table 2:** Correlations between inflammatory markers, adiponectin, and the components of metabolic syndrome.

	hs-CRP (mg/L)	IL-6 (pg/mL)	Adiponectin (µg/mL)
	*r* 1 (*p* values)		
Systolic blood pressure (mmHg)	0.11 (0.14)	0.12 (0.14)	−0.08 (0.34)
Diastolic blood pressure (mmHg)	0.26 (<0.01)	0.20 (0.01)	−0.28 (<0.01)
Waist circumference (cm)	0.47 (<0.01)	0.33 (<0.01)	−0.30 (<0.01)
Fasting glucose (mmol/L)	0.27 (<0.01)	0.09 (0.26)	−0.25 (<0.01)
TG (mmol/L)	0.20 (0.01)	−0.03 (0.77)	−0.32 (<0.01)
HDL-C (mmol/L)	−0.26 (<0.01)	−0.22 (<0.01)	0.41 (<0.01)
hs-CRP (mg/L)	-	0.46 (<0.01)	−0.24 (<0.01)
IL-6 (pg/mL)	0.46 (<0.01)	-	−0.11 (0.06)

1 Correlation coefficient (n = 177). HDL-C, high density lipoprotein-cholesterol; hs-CRP, high sensitivity C-reactive protein; IL-6, interleukin-6; TG, triglyceride.

The correlations between the levels of inflammatory markers, adiponectin, an oxidative stress, and antioxidant enzymes activities are shown in Table 3. The level of hs-CRP was significantly correlated with the level of MDA (*r* = 0.13, *p* = 0.01), and with the activities of CAT (*r* = −0.10, *p* = 0.07), SOD (*r* = −0.16, *p* = 0.04) and GPx (*r* = −0.15, *p* = 0.04). The level of IL-6 was significantly correlated with the activities of SOD (*r* = −0.17, *p* = 0.04) and was slightly correlated with the activities of GPx (*r* = −0.10, *p* = 0.08). The level of adiponectin was significantly correlated with the activities of GPx (*r* = 0.12, *p* = 0.03).

**Table 3:** Correlations between inflammatory markers, adiponectin, oxidative stress, and antioxidant enzymes activities.

	hs-CRP (mg/L)	IL-6 (pg/mL)	Adiponectin (µg/mL)
	*r* 1 (*p* values)		
MDA (µM)	0.13 (0.01)	0.05 (0.56)	−0.05 (0.52)
CAT (U/mg protein)	−0.10 (0.07)	−0.07 (0.39)	0.04 (0.61)
SOD (U/mg protein)	−0.16 (0.04)	−0.17 (0.04)	0.01 (0.87)
GPx (U/mg protein)	−0.15 (0.04)	−0.10 (0.08)	0.12 (0.03)

1 Correlation coefficient (n = 177). CAT, catalase activity; GPx, glutathione peroxidase; hs-CRP, high sensitivity C-reactive protein; IL-6, interleukin-6; MDA, malondialdehyde; SOD, superoxide dismutase

We calculated the ORs of MS based on the levels of inflammatory markers and adiponectin (Table 4). Subjects with higher levels of hs-CRP (≥1.00 mg/L) and IL-6 (≥1.50 pg/mL) or a lower level of adiponectin (<7.90 µg/mL) had a significantly greater risk of MS after adjusting for age, gender, and triglyceride level.

**Table 4:** The odds ratios of metabolic syndrome based on the levels of inflammatory markers and adiponectin.

	Odds ratio (95% CI)	*p* value
hs-CRP <1.00 mg/L	1.00	-
hs-CRP ≥1.00 mg/L		
Model 1^1^	2.22 (1.20–4.12)	0.01
Model 2^2^	2.42 (1.25–4.65)	<0.01
Model 3^3^	2.39 (1.16–4.94)	0.02
IL-6 <1.50 pg/mL	1.00	-
IL-6 ≥1.50 pg/mL		
Model 1	2.40 (1.13–5.11)	<0.01
Model 2	2.01 (1.14–3.55)	0.02
Model 3	2.73 (1.08–6.94)	0.04
Adiponectin ≥7.90 µg/mL	1.00	-
Adiponectin <7.90 µg/mL		
Model 1	2.25 (1.02–4.96)	0.04
Model 2	3.12 (1.20–8.13)	0.02
Model 3	3.46 (1.20–9.99)	0.02

1 None adjusted. ^2^Adjusted for age and gender. ^3^ Adjusted for age, gender, and triglyceride.

CI, Confidence interval; hs-CRP, high sensitivity C-reactive protein; IL-6, interleukin-6.

## Discussion

The present study showed a statistically significant link between the levels of inflammatory markers, adiponectin, and oxidative stress in MS. Subjects with MS had higher inflammation statuses (Figure 1). A variety of features of the metabolic syndrome are associated with systemic inflammatory responses [25]. In present study, we observed that the levels of inflammatory markers (hs-CRP and IL-6) were significantly positively correlated with the components of MS; in contrast, the level of adiponectin was inversely correlated with the components of MS. (Table 2). We calculated the ORs of MS according to the levels of inflammatory markers and adiponectin (Table 4). The level of hs-CRP (1.0 mg/L) or IL-6 (≥1.5 pg/mL) was used as a cut-off point to define higher inflammation status, which is an average risk factor for coronary artery disease [25], [26]. Plasma level of adiponectin in human is substantially high, up to 5 to 10 µg/mL on average [18]. In the present study, we have tried to calculate the ORs of MS according to the 50^th^ percentile of adiponectin level (5.80 µg/mL), there was no significant correlation between this level of adiponectin and the risk of MS. As a result, the 75^th^ percentile of adiponectin level (7.90 µg/mL) was used as a cut-off point in present study. Subjects with a higher inflammation status (hs-CRP ≥1.0 mg/L, IL-6 ≥1.50 pg/mL or adiponectin <7.90 µg/mL) had a significantly increased risk of MS. It seems that chronic inflammation is part of MS [27], [28] and that inflammatory markers (hs-CRP and IL-6) and adiponectin are significant risk factors for MS. We suggested the levels of inflammatory markers (hs-CRP and IL-6) and adiponectin could be incorporated in the diagnostic biomarkers for MS.

Oxidative stress is thought to play an important role in the development of MS [9]. Although the level of MDA was not significantly different between the case and control groups, the activities of CAT, SOD, and GPx were significantly lower in the case group (Figure 2). In the present study, we also assessed the correlations between inflammatory markers and oxidative stress makers. We observed that the inflammatory markers were significantly correlated with increased oxidative stress (Table 3). In particular, subjects with higher inflammation status (hs-CRP ≥3.0 mg/L) had significantly higher MDA level and lower antioxidant enzymes activities (data not shown). There was a significant positive correlation between inflammation status and oxidative stress, and we presume that subjects with MS may have a higher inflammation status and a higher level of oxidative stress. Antioxidant enzymes are the first line of defense against ROS and lead to a decrease in their activities [12]. In addition, MS subjects in general were typically abdominally obese. In the present study, we observed that the value of waist circumference was significant correlated with SOD (*r* = −0.25, *p*<0.01) and GPx activities (*r* = −0.41, *p*<0.01), respectively. The ratio of waist to hip was significantly negative correlated with SOD (*r* = −0.22, *p*<0.01) and GPx activities (*r* = −0.30, *p*<0.01), respectively. The values of body mass index were significantly negative correlated with GPx activities (*r* = −0.34, *p*<0.01). The values of waist circumferences were significantly positive correlated with the level of MDA in the case group (*r* = 0.24, *p* = 0.047). Obesity is an oxidative burden that may lead to the reduction of antioxidant enzymes activities [29], and induced inflammation plays a pathogenic role in the development and progression of MS [30].

Our study has some limitations. First, the number of participants was small, although we did recruit more subjects than we expected to recruit. Second, this study was a cross-sectional study, and therefore, no causal relationship could be defined. Larger, prospective studies are needed to establish the relationship between inflammation and oxidative stress in MS patients. Third, we selected MDA as a oxidative stress marker and hs-CRP and IL-6 as inflammatory markers in the present study; further studies could select more sensitive markers of oxidative stress such as urinary 8-epi-prostaglandin F_2α_, plasma oxidized LDL or the ratio of reduced glutathione to oxidized glutathione and other important adipokines (such as tumor necrosis factor α, IL-1β, leptin, monocyte chemoattractant protein-1 or proteins of the renin angiotensin system) in subjects with MS.

In conclusion, subjects suffering from MS may have a higher inflammation status and a higher level of oxidative stress. A higher inflammation status was significantly correlated with lower the levels of antioxidant enzymes and adiponectin, and greater risk of MS.
